# Combined assay of Circulating Tumor DNA and Protein Biomarkers for early noninvasive detection and prognosis of Non-Small Cell Lung Cancer

**DOI:** 10.7150/jca.49647

**Published:** 2021-01-01

**Authors:** Jian-xin Yin, Wen-wei Hu, Hua Gu, Jian-min Fang

**Affiliations:** 1 School of Life Sciences and Technology, Tongji University, Shanghai, China.; 2 Shanghai Tongji Hospital, Tongji University, Shanghai, China.; 3 Tongji University Suzhou Institute, Suzhou, Jiangsu, China.

**Keywords:** non-small cell lung cancer, next-generation sequencing, cancer early detection, circulating tumor DNA, tumor biomarkers

## Abstract

**Purpose:** Early diagnosis of lung cancer is critical to curtailing cancer-related deaths. We aimed to develop a highly sensitive assay for the analysis of circulating tumor DNA (ctDNA) to detect non-small cell lung cancer (NSCLC) in the early stages.

**Materials and Methods:** We detected *EGFR* and *KRAS* mutations in paired plasma and tumor tissue samples from 147 NSCLC patients. Of these, *EGFR*/*KRAS* ctDNA mutations and protein biomarkers were comparatively analyzed in 87 individuals. In addition, tissue samples of 20 patients were subjected to repeat multi-gene detection, and pre- and post-operative paired samples of 28 patients were subjected to multi-gene detection. Clinical information was obtained to complement the prognostic value of the combined assay results and post-operative new ctDNA mutation status.

**Results:** *EGFR*/*KRAS* mutations were highly consistent in ctDNA and tumor DNA. Combining the detection of *EGFR* and *KRAS* mutations in ctDNA with the detection of protein biomarkers increased cancer detection sensitivity to 74.7% (65/87). None of the healthy controls tested positive using the combined assay (100% specificity). Combined assay results independently associated with recurrence-free survival. Post-operative new ctDNA mutation status independently associated with overall survival and recurrence-free survival.

**Conclusion:** The detection of ctDNA may be exploited for early diagnosis of NSCLC, as highlighted by the developed assay. Further, the combined assay results and post-operative new ctDNA mutation status are promising prognostic indicators in NSCLC patients.

## Introduction

Lung cancer is the leading cause of cancer-related mortality worldwide 1. Non-small cell lung cancer (NSCLC) represents a major histological class of lung cancer, accounting for approximately 85% of all cases. Currently, most NSCLC cases in China are detected based on clinical symptoms, such as persistent cough, chest pain, and hemoptysis, rather than by lung cancer screening. Unfortunately, during early-stage lung cancer, affected individuals are asymptomatic, and 75% of individuals are diagnosed at the advanced stage of cancer. Compared with the 5-year survival rate of 70-80% for stage IA NSCLC, the 5-year survival rate of stage IIIA is only 24% 2. Consequently, early diagnosis is critical for prolonging the survival of individuals with NSCLC.

Protein biomarkers, whose serum levels are elevated in individuals with cancer, play an important role in tumor diagnosis 3. However, pregnancy, smoking, inflammation, and some benign diseases might also lead to an increase in the serum levels of certain protein biomarkers. In recent years, liquid biopsies have increasingly received attention. Among them, circulating tumor DNA (ctDNA) is a very widely used biomarker.

It is well known that due to tumor heterogeneity, the mutations harbored by tumor cells in different parts of the same tumor or different metastases may differ 4, further complicating targeted drug selection. Genetic testing of a single tissue sample often fails to reflect the complete mutation spectrum of the disease. Hence, drug resistance may appear soon after the introduction of sub-optimal targeted drugs 5, affecting the survival rate. Derived from all tumor cells, ctDNA fully reflects the mutation spectrum of the disease, which is crucial for the selection of treatment strategy.

Advances in ctDNA research highlight the potential applications of liquid biopsies for the detection and management of cancer patients. Compared with tissue biopsies, the liquid biopsies are noninvasive, and allow easy and repeated sampling before and after the surgery, which facilitates the monitoring of disease evolution 6, minimal residual disease 7, and response to therapy 8.

Nevertheless, ctDNA analysis has some limitations. In peripheral blood, ctDNA is highly diluted by cell-free DNA (cfDNA) from healthy cells, with a half-life of approximately 2 h 9. The detection of minute amounts of ctDNA, especially in early-stage cancer patients, is challenging because of the background noise from cfDNA 10. Further, the mechanism of ctDNA release by tumor cells remains unclear. The time interval between two ctDNA molecules containing the same mutation into peripheral blood and the time between the occurrence of a mutation to when it is present within ctDNA affects the clinical value of ctDNA analysis in practice.

Extremely sensitive detection methods have been developed to detect low-frequency mutations in early-stage tumor patients 11, 12. Mouliere *et al.* analyzed the length of ctDNA fragments in tumor patients in detail and found that it was 20-40 bp shorter than the normal cfDNA 13. The sensitivity of their assay can be increased by *in vitro* and *in silico* size selection of 90-150 bp cfDNA fragments. Different researchers have deployed various approaches to ctDNA detection. For example, Shen *et al.* developed a bisulfite-free methodology for immunoprecipitation-based profiling of methylation patterns in cfDNA 14, which was not restricted by the limited number of recurrent mutations 12, 15. Cristiano *et al.* developed an approach for evaluating the fragmentation patterns of cfDNA across the genome and found that profiles of healthy individuals are significantly different from those of patients with cancer (57-99% sensitivity across seven tumor types) 16. Nevertheless, many mutations are common to different cancers, and detection of mutation alone cannot be used to infer the anatomical location of the primary tumor. As in another study, Cohen *et al.* combined the detection of ctDNA and quantitation of tumor-associated protein biomarkers to detect and locate eight cancer types 17. However, the detection sensitivity of lung cancer is lower than 60%.

In the current study, we established a next-generation sequencing (NGS) assay based on polymerase chain reaction (PCR) for ctDNA detection in individuals with NSCLC. We tested the assay by performing a retrospective study using material from NSCLC patients. We show that the assay is highly specific and sensitive. Combining the ctDNA analysis with protein biomarker detection improved the sensitivity of NSCLC detection. Expanding the gene panel assayed likewise improved the detection sensitivity. The combined assay may be used for the detection and prognosis of NSCLC patients. Notably, the current strategy may be applied to the analysis of other cancer types.

## Materials and Methods

### Samples collection

For the study, 147 NSCLC patients and 25 healthy individuals without cancer were enrolled at the Pulmonary Hospital in Shanghai (China) from December 2011 to March 2013. Informed written consent was obtained from all patients and healthy individuals before the study. The demographic, pathological, and clinical data for all patients were retrieved from clinical reports, and they are summarized in Table 1. Tumor tissues were obtained during the surgery. The peripheral blood samples were collected 1 d before the surgery and 5 d after the surgery.

### DNA extraction from tumor and blood samples

Tumor genomic DNA of fresh-frozen tumor tissue and leukocytes genomic DNA of healthy individuals were extracted using the TIANamp Genomic DNA Kit (Tiangen, Beijing, China), following the manufacturer's instructions. The concentration of genomic DNA was measured by using Nanodrop 2000 (Thermo Fisher Scientific, Wilmington, DE).

The blood was collected in 10-ml K2 EDTA vacutainer tubes (BD Vacutainer, Franklin Lakes, NJ), and the plasma was isolated within 2 h of blood collection. The tubes were centrifuged at 1600×*g* for 10 min at 4 °C, and the plasma was further transferred to 1.5 ml microcentrifuge (Eppendorf) tubes and centrifuged at 16,000×*g* for 10 min at 4 °C. The supernatant was collected and stored at -80 °C until cfDNA extraction. The cfDNA was extracted using the Serum/Plasma Circulating DNA Kit (Tiangen, Beijing, China). The Qubit^®^ 3.0 Fluorometer and Qubit dsDNA HS Assay kit (Life Technologies, Carlsbad, CA) were used to quantify cfDNA, following the manufacturer's protocol.

### NGS library construction

The analysis was performed using two ultra-deep targeted NGS panels: panel one for *EGFR* and *KRAS* (6 amplicons covering 933 bases) (Table S1); and panel two for the analysis of recurrent mutation regions of selected lung cancer-association genes (19 amplicons covering 2096 bases across 11 genes) (Table S2).

The NGS libraries were prepared using target-specific exponential amplification and barcoding PCR. In the first round of amplification, amplification primers were used. The primer pairs were pooled, and their concentrations adjusted according to the amplification efficiency. Round 1 amplification was performed using Q5 HiFi PCR Mastermix (NEB, Beijing, China) with the following cycling conditions: 98 °C for 30 s; followed by 15 cycles at 98 °C for 10 s and 62 °C for 15 s; followed by incubation at 72 °C for 5 min. The product from round 1 amplification was used as the template for the round 2 amplification. In the second round of amplification, fusion primers consisting of sequencing primers, barcodes, and adaptor sequences were used to introduce sample-specific barcodes and sequencing adaptors to library sequences (Figure S1). Round 2 amplification was performed using Q5 HiFi PCR Mastermix (NEB, Beijing, China), with the following cycling conditions: 98 °C for 30 s; followed by 20 cycles at 98 °C for 10 s and 60 °C for 15 s; followed by incubation at 72 °C for 5 min. The intermediate and final PCR products were purified by using Hieff NGS^®^ Smarter DNA Clean Beads (Yesen, Shanghai, China) at 1.6× volume ratio of magnetic beads to PCR products, and the products were eluted in 20 μL of PCR-grade water. For the multi-gene panel, round 1 and round 2 of PCR were performed, whereas for the *EGFR*/*KARS* panel, only round 2 of PCR was performed. In round 2, we constructed a forward sequencing library and a reverse sequencing library for each sample.

The forward and reverse sequencing libraries of 25 samples were mixed into a single sequencing library. The library was purified using AMPure beads (Beckman Coulter, Brea, CA), quantified using Qubit 3.0 fluorometer (Life Technologies), and sequenced using the Ion Proton^TM^ System (Life Technologies, CA, USA). Sequencing data were processed and analyzed using the Torrent Suite v5.2.1 (Life Technologies, USA). The Ion Reporter and Integrative Genome Viewer v2.6.3 18 were used for variant annotation and read visualizations, respectively.

### Variant calling

To determine the minimum variant allele frequency (VAF) threshold, two DNA fragments were synthesized (Table S3). One fragment contained three specific mutations, while the other was a wild-type sequence. The mutated fragment was mixed with the wild-type fragment at a ratio of 1:1000. After amplification and sequencing, the results revealed that a specific mutation could be detected at a 0.5% VAF threshold, while the false-positive mutations were excluded. Considering the extremely low frequency of mutations in healthy individuals and accuracy of the Ion Proton^TM^ System, a 0.5% threshold was set. The sequence data were filtered by using the Torrent Suite Software v3.0. The adapter sequences, short segments, and low-quality segments were excluded. Next, “variant caller v5.0” plug was used to analyze the mutation, using the following criteria: average coverage depth > 8000; each variant coverage > 20, *p* < 0.01; each VAF > 0.5%. The ratio of forward and reverse mutations (*R1*) to the ratio of forward and reverse sequencing depth (*R2*) ranged between 0.5 and 2 (0.5 < *R1* / *R2* < 2). The Integrative Genome Viewer software v2.6.3 was used to examine the sequencing error and manually identify the missing mutations. Finally, the Catalogue of Somatic Mutations in Cancer database was used to validate these mutations.

### Statistical analysis

McNemar's test was used to compare the mutation difference between paired plasma and tissue tests. The Kappa statistic is used to assess the mutation consistency between the paired plasma and tissue tests. The *t*-test was used to compare the VAF of mutations found in both ctDNA and lymph nodes. Wilcoxon matched-pairs signed ranks test was used to compare the mutation status of repeat tissue sequencing with the custom panel, and the difference between pre- and post-operative mutations. Data analyses were performed using SPSS version 22.0 (IBM, Armonk, NY) and GraphPad Prism 8.0 (GraphPad, La Jolla, CA). Survival analysis was performed using the Kaplan-Meier method. The Cox proportional-hazards regression model was used for univariate and multivariate analysis. Survival analysis was performed using the “survival” and “survminer” packages in R version 3.4.3. The *p*-value < 0.05 was considered statistically significant.

## Results

### EGFR and KRAS analysis for NSCLC detection

*EGFR* and *KRAS* as oncogenes are closely related to the occurrence and progression of NSCLC. *EGFR* and *KRAS* mutations rank first in gene mutation frequency among Asian and Euro-American populations, respectively. In the current study, an NGS panel targeting exons 18, 19, 20, and 21 of *EGFR*, and exons 2 and 3 of *KRAS* was used to compare the mutation status of ctDNA and paired tumor tissue DNA (tDNA). The study material was obtained from 147 individuals with stages I to IV NSCLC. Their histopathological and clinical characteristics are summarized in Table 1.

**Figure 1:**
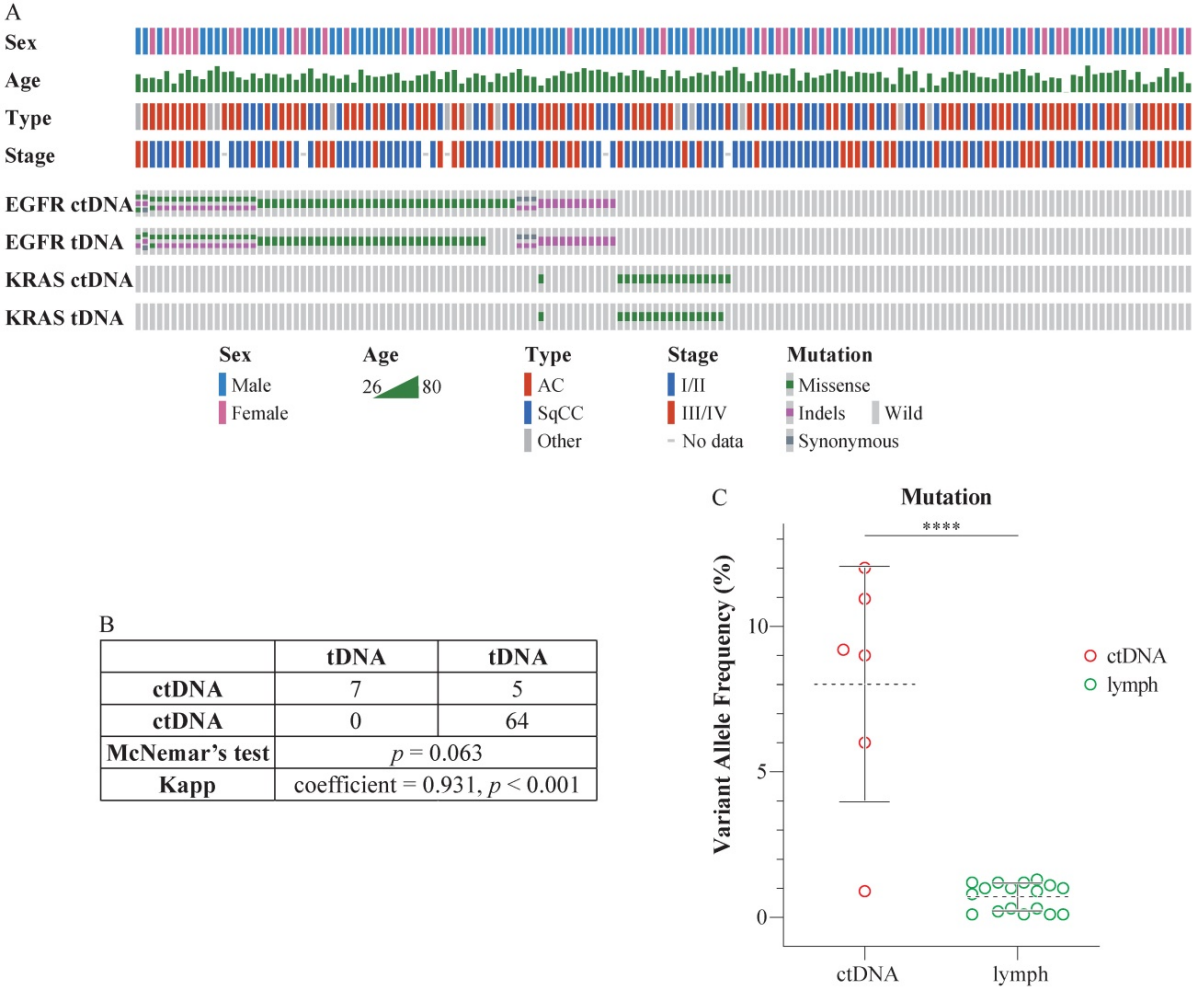
Comparison of mutations in ctDNA and tDNA from NSCLC patients. (A) The mutation status of *EGFR* and *KRAS* in the peripheral blood and tissues of 147 patients. Each column represents one patient. The number of colored blocks on the gray bar indicates the number of mutations. (B) Comparison of plasma and tissue tests. McNemar's test, *p* > 0.05; Kappa statistic = 0.931, *p* < 0.001. (C) Comparison of VAF in ctDNA and the lymph nodes. Dotted lines indicate the mean VAF of mutation in ctDNA and the lymph nodes; whiskers represent standard deviations;* t-*test, two-tailed, ****p < 0.0001.

The sequencing depth exceeded 8000×, which qualified the data for the detection of ctDNA mutations. Eighty-three patients harbored mutations in *EGFR* (67 cases) or *KRAS* (17 cases) in ctDNA. Specifically, 19.28% (16/83) of these individuals harbored two *EGFR* mutations; 1.2% (1/83) harbored three *EGFR* mutations; and 1.2% (1/83) harbored mutations in both *EGFR* and *KRAS*. Seventy-eight patients harbored mutations in *EGFR* (63 cases) or *KRAS* (16 cases) in tDNA. In particular, 21.80% (17/78) of these individuals harbored two *EGFR* mutations, and 1.28% (1/78) harbored mutations in both *EGFR* and *KRAS*. Detailed information is presented in Figure 1A (prepared using Oncoprinter 19, 20) and Table S4. There were no significant differences between the ctDNA and tDNA tests (McNemar's test, *p* > 0.05), and the assays were highly consistent (Kappa statistic = 0.931, *p* < 0.001) (Figure 1B).

The mutation status of ctDNA and tDNA was different in six patients. *EGFR* V769L, *EGFR* T790M, *EGFR* L858R, *EGFR* L858R, *EGFR* S768I, and *KRAS* G12D mutations were detected in the ctDNA from case 38, 95, 109, 117, 119, and 137, respectively. However, no corresponding mutations were detected in their tDNA. Subsequently, we performed additional sequencing of the five lymph nodes surrounding the tumor, resected during the surgery, for each of the six patients. The mutations in their respective ctDNA were detected in a portion of the lymph nodes. Besides, the VAF in lymph nodes was significantly lower than VAF in ctDNA (Figure 1C).

### Combined assay of ctDNA and protein biomarker and clinical value of combined assay in prognosis

We next attempted to combine the detection of ctDNA with the serum levels of protein biomarkers to increase the sensitivity of tumor diagnosis. We focused on the carcinoembryonic antigen (CEA), fragments of cytokeratin 19 (CYFRA21-1), and squamous cell carcinoma antigen (SCC), biomarkers extensively used in NSLSC diagnosis.

The level of protein biomarkers' specificity mainly depends on the cutoff value. In the current study, we established the cutoff values for CEA, CYFRA21-1, and SCC at 7.3 ng/mL, 4 ng/mL, and 2.5 ng/mL, respectively, based on previous reports 3, 21-24. These values are all above the clinical standard cutoff level (5 ng/mL, 3.3 ng/mL, and 1.5 ng/mL, respectively).

**Figure 2:**
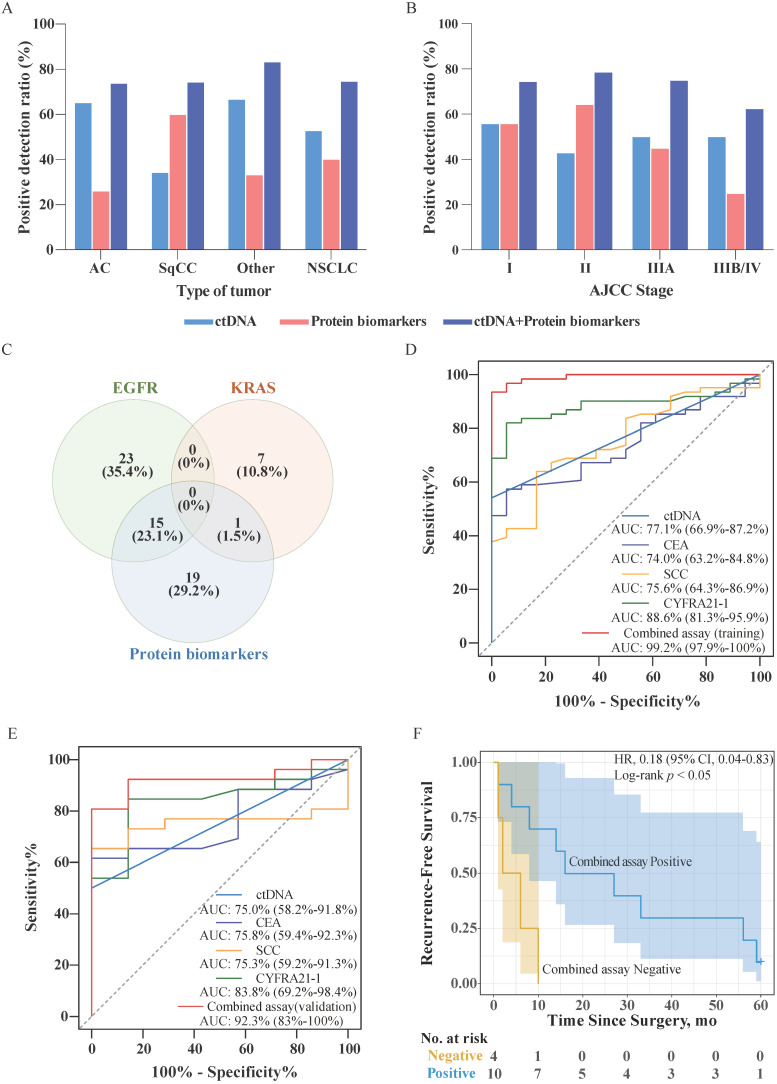
** Combined assay in NSCLC patients.** (A) Sensitivities of approaches involving stand-alone ctDNA analysis, stand-alone protein biomarker analysis, and combined assay in different tumor types. (B) Sensitivities of approaches involving stand-alone ctDNA analysis, stand-alone protein biomarker analysis, and combined assay in different AJCC stages. (C) Venn diagram of the number and proportion of cancer patients identified based on *EGFR* mutation, *KRAS* mutation, and protein biomarkers. (D) Receiver operator characteristic (ROC) curves for ctDNA, CEA, SCC, CYFRA21-1, and the combined assay using the training data. The area under the curve (AUC; 95% confidence interval) represents the detection performance of the different indicators. (E) Receiver operator characteristic (ROC) curves for ctDNA, CEA, SCC, CYFRA21-1, and the combined assay using the validation data. (F) Kaplan-Meier estimates of recurrence-free survival for 14 patients with AJCC Stage III/IV NSCLC stratified using combined assay results. Shaded areas represent 95% CIs. HR: hazard ratio; CI: confidence interval.

We have analyzed data for 87 NSCLC patients and 25 healthy individuals, whose biochemical test reports were available (Table S5). Accordingly, 43.7% (38/87) of patients harbored *EGFR* mutation in ctDNA, while 9.2% (8/87) of patients harbored *KRAS* mutations in ctDNA. In 40.2% (35/87) of patients, the level of at least one protein biomarker was higher than the corresponding cutoff value. By contrast, no elevated serum levels of biomarkers were detected in 25 healthy individuals. In addition, no *EGFG*/*KRAS* mutations were detected in the genomic DNA purified from leukocytes from healthy individuals. The detailed distribution of ctDNA mutations and protein biomarkers found in NSCLC patients is presented in Table 2. The positive detection ratios of ctDNA mutations, protein biomarkers, and in a combined assay are depicted in Figure 2A, and Figure 2B, according to the type of NSCLC and American Joint Committee on Cancer (AJCC) stage, respectively. As shown in Figure 2A, the ctDNA detection ratio was higher in adenocarcinoma (AC) (65.2%) and other tumor type (66.7%) than that in squamous cell carcinoma (SqCC) (34.3%), while the positive detection ratio for protein biomarkers in SqCC (60.0%) was better than that in AC (26.1%) and other types (33.3%). As shown in Figure 2B, the positive detection ratios of ctDNA mutations in Stage I were higher than in the other stages. The positive detection ratios of protein biomarkers in Stage I and II were higher than those in Stage IIIA and IIIB/IV. Additionally, the positive detection ratios of the combined assay in Stage I, II, IIIA were higher than Stage IIIB/IV, whereas in AC and SqCC were equal.

The proportions of patients testing positive for *EGFR* and *KRAS* mutations in ctDNA and protein biomarkers are shown in Figure 2C. The analysis revealed that the presence of mutations in *EGFR* and *KRAS* were mutually exclusive in the sampled population. Further, only 24.6% (16/65) of patients were positive for both, ctDNA mutations and protein biomarkers. However, a larger proportion of patients tested positive for either the presence of ctDNA mutations or protein biomarkers (46.2% and 29.2%, respectively). In the combined assay, the patients were considered for testing positive if their ctDNA harbored mutations in either *EGFR* or *KRAS*, or if the serum level of any protein biomarker exceeded the threshold. Consequently, the positive detection rate of the combined assay increased to 74.7% (65/87) without decreasing the specificity of the assay.

To assess the performance of each biomarker for NSCLC, we used the receiver operator characteristic (ROC) curve analysis. We randomized patients into a training and validation group and analyzed ctDNA, CEA, SCC, and CYFRA21-1 using the training data (Figure 2D). For the combined assay (training group), we first established a regression model using a binary logistic regression analysis of the biomarkers and then performed the ROC curve analysis (Figure 2D). The analysis indicated that the detection performance of the combined assay in NSCLC was higher than that of the performance of the individual biomarkers. We then tested the regression model using the validation set data and performed a ROC analysis (Figure 2E). The results showed that the results of the analysis using the validation set data were consistent with the results of the training set, indicating that the regression model was successfully constructed.

In order to evaluate the difference in terms of recurrence-free survival (RFS) of patients with positive or negative combined assay results, we followed up with these 87 patients for 5 years. Among them, RFS data were available for a subset of 42 patients. Survival analysis showed that among Stage III/IV patients (33.3%, 14/42), the combined assay results indicated that negative individuals have significantly poorer RFS than the positive individuals (Figure 2F). However, among Stage I/II patients, there was no significant difference between combined assay results positive and negative individuals (*p* > 0.05). Subsequently, the Cox proportional hazards regression analysis was used to assess the association of combined assay results and clinicopathological factors with RFS. As shown in Table 3, univariate analysis showed that the AJCC Stage was significantly associated with RFS (*p* < 0.001), and had a trend for combined assay results (*p* = 0.087). Further, the AJCC Stage and combined assay were fitted in the Cox proportional hazards regression model. The multivariate analysis revealed that the combined assay results and AJCC Stage were independent prognostic indicators for the RFS (*p* = 0.012, and *p* < 0.001, respectively).

### Reproducibility testing of multi-gene detection

Increasing the number of analyzed genes can increase the sensitivity of tumor detection. Therefore, we next used a large custom panel (Table S2) to detect NSCLC, and compared the results with those of the *EGFR/KRAS* panel.

Evaluation of the detection reproducibility of the custom panel involved parallel sequencing of tDNA from the same sample. The ctDNA copy number in the plasma is low, which may affect the consistency with which the mutations are detected. By contrast, the quantity of DNA extracted from tumor tissue was sufficient for analysis. Therefore, in the current study, we used tissue DNA for the ensuing analysis.

**Figure 3:**
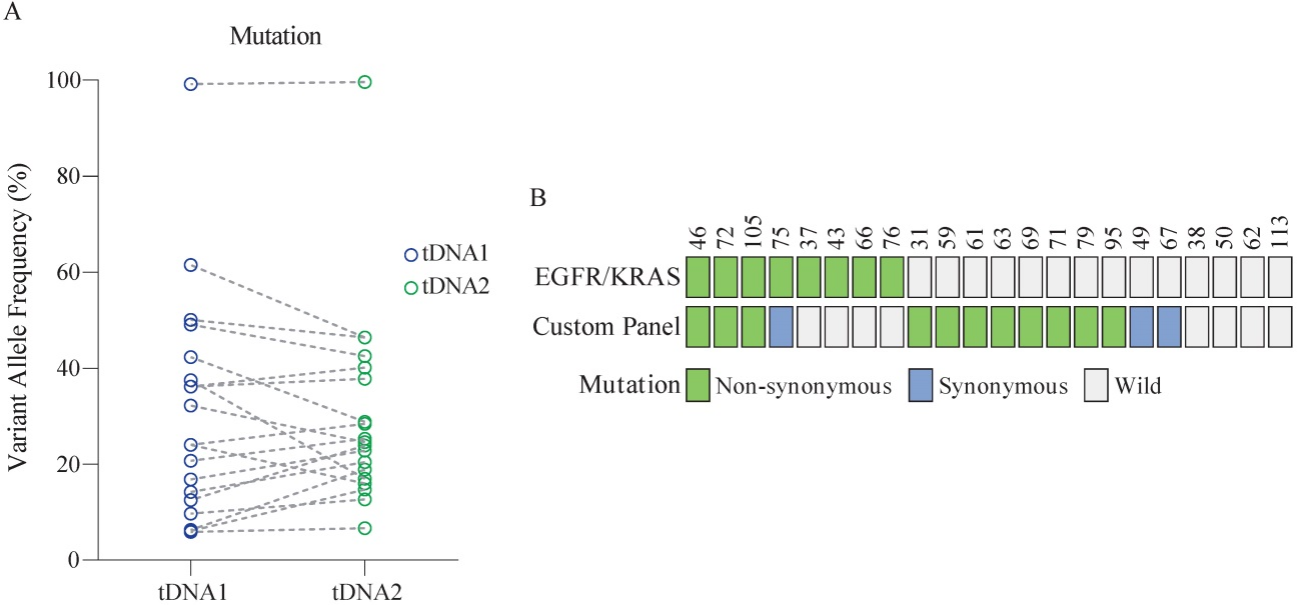
** Performance of the custom panel.** (A) Comparison of VAF in tDNA detected by two sequencing runs (tDNA1 and tDNA2) using the custom panel. Wilcoxon matched-pairs signed-rank test, two-tailed, *p* > 0.05. (B) Comparison of the performance of the custom panel and *EGFR*/*KRAS* panel. Each column represents one patient.

Twenty-two patients aged (46-70 years) were randomly enrolled from the 147 patients for the analysis. The mutation status in the analyzed samples of tissue DNA was identical, with no significant difference in the VAF between samples (Figure 3A, Table S6). Further, at least one mutation detected using the custom panel was validated in 86.4% (19/22) of patients (Figure 3B). Among these, 63.2% (12/19) of patients harbored non-synonymous mutations. By comparison, only 30% of patients were identified as positive for the *EGFR* or *KRAS* mutation. Overall, the sensitivity of the two panels combined was up to 86.4% (19/22).

### Response of ctDNA to therapy and clinical value of ctDNA in prognosis

The number and VAF of mutations in ctDNA changes in real-time with the occurrence, progression, and treatment of disease. Therefore, we next investigated the response of ctDNA to surgical treatment.

**Figure 4:**
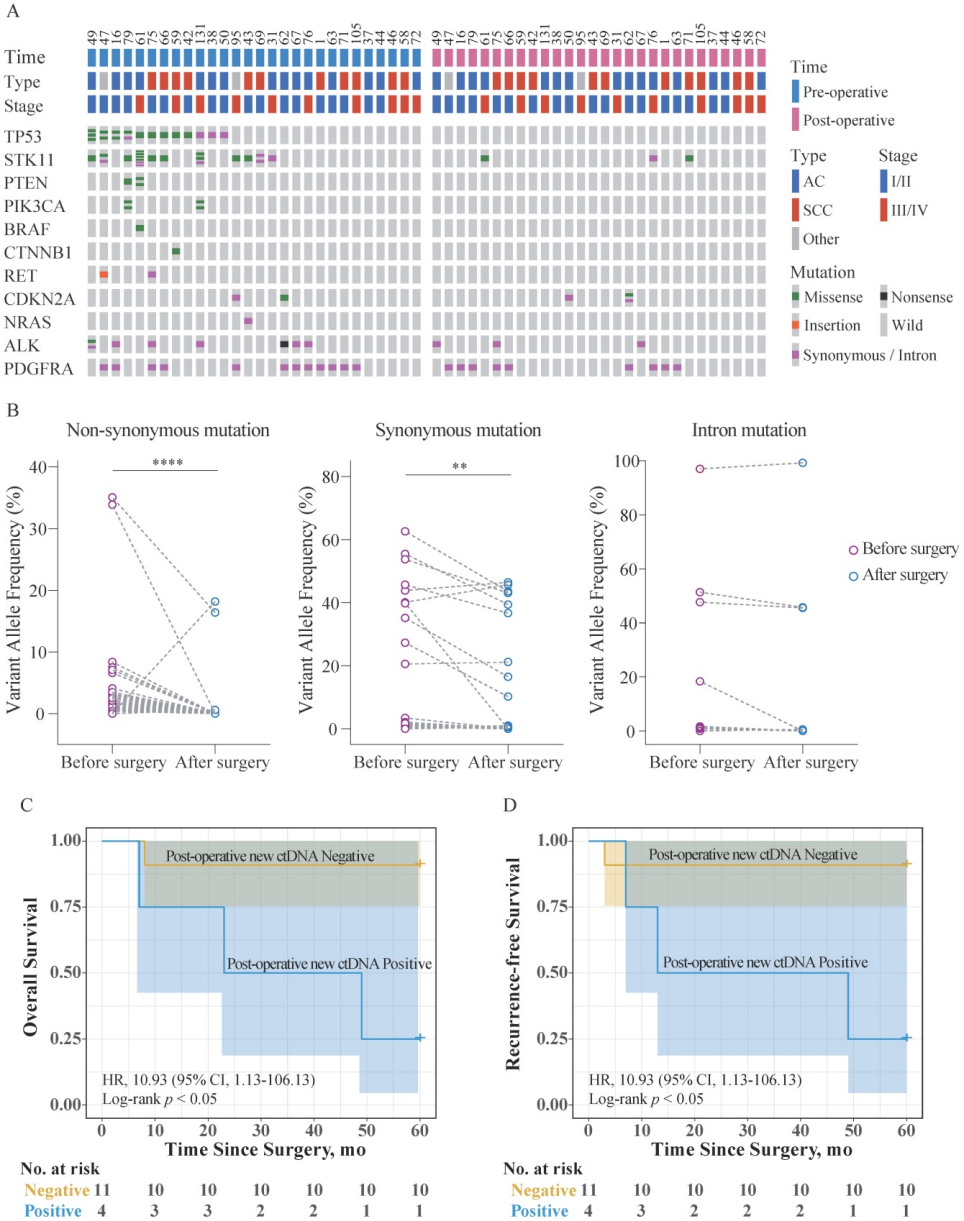
** Clinical application of the custom panel.** (A) Comparison of the mutation status of ctDNA 1 d before the surgery and 5 d after the surgery using the custom panel. Each column represents one patient. The number of colored blocks on the gray bar indicates the number of mutations. (B) Comparison of VAF in ctDNA obtained 1 d before the surgery and 5 d after the surgery. Wilcoxon matched-pairs signed-rank test, two-tailed, **** *p* < 0.0001 and ** *p* < 0.01. (C) Kaplan-Meier estimates of overall survival for 15 patients with AJCC Stage I/II NSCLC stratified using post-operative new ctDNA mutation status. (D) Kaplan-Meier estimates of recurrence-free survival for 15 patients with AJCC Stage I/II NSCLC stratified using post-operative new ctDNA mutation status. Shaded areas represent 95% CIs. HR: hazard ratio; CI: confidence interval.

Twenty-eight patients aged (34-74 years) were randomly selected from the 147 patients for the analysis. The ctDNA was analyzed both, 1 d before the surgery and 5 d after the surgery. The mutation and clinical data for these patients are shown in Figure 4A, Table S7, and Table S8. Notably, the number of non-synonymous mutations in ctDNA (missense, nonsense, and insertions) was expectedly reduced after the surgery. Comparison of the VAF in ctDNA obtained at the two different timepoints revealed that synonymous mutations, non-synonymous mutations, and mutations in introns showed different trends (Figure 4B). VAF of the intron mutations did not change significantly, while VAF of the non-synonymous and synonymous mutations significantly decreased, to varying degrees.

These patients were divided into two groups according to whether new ctDNA mutations occurred after surgery, and they were followed up for 5 years. Among them, overall survival (OS) and RFS data were available for 20 patients. Survival analysis showed that among Stage I/II patients (75.0%, 15/20), the post-operative new ctDNA mutation-positive individuals have significantly poorer OS and RFS than the negative individuals (Figure 4C, Figure 4D). No Stage III/IV patients were post-operative new ctDNA mutation-positive. Further, the Cox proportional hazards regression analysis was used to assess the association of post-operative new ctDNA mutation status and clinicopathological factors with OS and RFS. As shown in Table 4 and Table 5, univariate analysis showed that AJCC Stage was significantly associated with OS (*p* = 0.023) and RFS (*p* = 0.045), respectively. Further, the AJCC Stage and the post-operative new ctDNA mutation status were fitted in the Cox proportional hazards regression model. The results of the multivariate analysis revealed that AJCC Stage and the post-operative new ctDNA mutation status were independent prognostic indicators for the OS (*p* = 0.013, and *p* = 0.042, respectively) and RFS (*p* = 0.020, and *p* = 0.038, respectively).

## Discussion

In the current study, we simultaneously detected *EGFR* and *KRAS* mutations in plasma and tissue samples of patients with NSCLC. We also tested the utility of the assay combined with protein biomarker detection and that of a broader, custom panel, to improve the sensitivity of NSCLC detection. The presented NGS-based combined assay approach is expected to be applied to the detection of multiple cancer types.

Bronchoscopy and surgical lung biopsies, when traditional imaging diagnosis suggests suspected cancer, are standard clinical examinations for lung cancer. However, these approaches cause trauma to the patient. In addition, there is a definite inherent risk associated with sample retrieval and the samples collected may not reflect an accurate portrayal of mutations present in different parts of the tumor in the patient. As in our cohort, six patients had mutations in ctDNA that were not present in their tDNA. However, the corresponding differential mutations were identified in some lymph nodes surrounding the respective primary tumors in these patients. Because of tumor heterogeneity, mutations in a part of the tumor tissue cannot reflect the complete mutation spectrum of the disease. Whereas ctDNA, which originates from all tumor cells, can make up for this deficiency. In the other 141 patients, the mutation status of *EGFR*/*KRAS* in ctDNA was consistent with that in tDNA. This demonstrated that *EGFR*/*KRAS* mutations in ctDNA were effective in reflecting the mutations identified in tDNA. If there is an insufficient amount of tumor tissue for molecular analysis or repeat testing should be required, ctDNA testing may be an alternative to tDNA testing. Although liquid biopsy supplements tissue biopsy to a certain extent, its potential value for clinical diagnosis should be verified by continuous practice.

In the current study, we showed that the *KRAS* and *EGFR* mutations in ctDNA are mutually exclusive (Figure 1A), which is consistent with previous studies 25. However, *EGFR* and *KRAS* co-mutation occurs in a few patients. In the current study, case 120 harbored both *EGFR* E746_A750del mutation and *KRAS* G12V mutation. This patient developed lymph node metastasis and relapsed after surgery. Li *et al.* reported that when *EGFR* and *KRAS* mutations do co-occur, the disease could be controlled by using drugs that target EGFR 26. This clinical case could be used as a reference for the treatment of co-mutation in multiple driver genes.

Protein biomarkers are widely used for the early diagnosis of lung cancer. However, because of the susceptibility to trauma, inflammation, and other diseases, the diagnostic utility of protein biomarkers is limited. The inclusion of the detection of tumor protein markers in routine physical examinations reflects the importance of tumor protein markers in the detection of cancer. The previous publications 27-29 overwhelmingly focus on ctDNA alone, while studies of ctDNA in combination with tumor protein markers are lacking. We here compared the utility of *EGFR* and *KRAS* ctDNA mutations with that of CEA, SqCC, and CYFRA21-1, biomarkers commonly used in the NSCLC diagnosis.

We first set a threshold for each biomarker that far exceeded that of the clinical test standard, to exclude individuals whose protein biomarker levels are elevated because of other (non-cancer-related) factors. The analysis revealed that the cancer detection sensitivity of ctDNA or protein biomarkers alone was only 52.9% and 40.2%, respectively, while the sensitivity of a combined assay was as high as 74.7%. Therefore, while the combined assay improved the detection sensitivity, it did not reduce the detection specificity. This notion was confirmed by the ROC curve analysis of the combined assay. Cohen *et al.* demonstrated the potential clinical utility of another combined assay for eight types of cancer detection simultaneously 17. Similarly, other biomarkers could be used in combination with ctDNA analysis to increase detection sensitivity.

Further, the survival analysis of 42 individuals revealed that among Stage III/IV patients, individuals with positive combined assay results had notably better survival expectations. This situation is closely related to studies on the carcinogenic mechanisms of *EGFR* and *KRAS* and the use of *EGFR*-targeted drugs. Meanwhile, the multivariate analysis results demonstrated the potential value of the combined assay as an RFS indicator.

In addition to combining with the analysis of protein biomarkers, improving the cancer detection sensitivity of ctDNA can also be achieved by increasing the number of genes probed. We here used a custom panel to repeatedly analyze 22 tissue samples from NSCLC patients. The detected mutation status was identical in the repeat analyses, with no significant difference in the VAF. This indicates that the proposed detection method is reliable and could be used for ctDNA detection. At the same time, the detection sensitivity of the custom panel was higher than that of the *EGFR*/*KRAS* panel. Notably, the sensitivity of the combined assay was as high as 86.4%.

The above findings highlight the advantages of multi-gene screening. Currently, the commercial caner panels often contain tens to hundreds of genes to allow for comprehensive analysis. Although this improves the detection sensitivity, identification of too many mutations may lead to unnecessary follow-up procedures, and anxiety in healthy individuals. According to Yizhak *et al.*, 33% of all individuals carry at least one non-synonymous mutation associated with cancer. Besides, an increased number of genes in a panel will inevitably increase the cost 30. Therefore, for a multi-gene detection of cancer, it is necessary to balance the detection sensitivity and economic factors. The lung cancer panel devised in the current study might allow more individuals to undergo early screening, which in turn would effectively reduce the number of deaths caused by cancer.

Although the short half-life of ctDNA poses a challenge for its detection, ctDNA analysis can be used to rapidly reflect the patient's mutation status and response to treatment. Comparison of mutations in ctDNA 1 d before the surgery and 5 d after the surgery revealed different trends for VAF of different mutation types. The findings suggested that non-synonymous mutations, the majority of synonymous mutations, and some intron mutations originate from tumor cells. The other mutations originate from healthy cells. At the same time, we observed new mutations in ctDNA 5 d after the surgery in samples from four patients. There are several possible sources of such new mutations detected in the blood 5 d after the surgery. First, following surgical resection of the tumor *in situ*, other metastatic lesions in the individual may release ctDNA under stress. These newly released ctDNA molecules would not be present in the peripheral blood collected prior to the surgery. Second, tumor cells in different parts of the tumor may carry different mutations 4. The ctDNA released by these tumor cells to the peripheral blood is relatively random, and the half-life of ctDNA is quite short 9. Therefore, the blood sample collected at one timepoint may not fully reflect the mutation status of all tumor cells. Third, the release of ctDNA caused by the destruction of tumor tissue during surgery cannot be ruled out, although the possibility of that happening is extremely low.

The survival analysis of 20 patients shown that among Stage I/II patients, the presence of new mutations in the ctDNA was associated with dramatically shorter OS and RFS. Further, the multivariate analysis results indicated the potential clinical value of the post-operative new ctDNA mutation status as OS and RFS indicators.

Some potential limitations of the current study should be acknowledged. For example, some mutations that were absent in the patient sample might have been artificially introduced in the first round of PCR. Since the primers used in round 1 of PCR lacked unique molecular identifiers, we could not distinguish whether the mutations were present in the original sample or artificially introduced. This technical issue persisted despite using Q5 High-Fidelity DNA Polymerases (NEB, Beijing, China). Further, the assay can only be used to detect single-nucleotide mutations or indels. Therefore, although we interrogate the mutations in *ALK* (using the custom panel), we are unable to evaluate the presence of *EML4*-*ALK* fusion because of the limits of the PCR-based targeted capture method.

In the future, DNA mismatch repair genes should be considered for gene panel design for tumor detection. Mutations in the mismatch repair genes lead to increased genomic instability and may result in a microsatellite instability phenotype. Le *et al.* demonstrated that patients with mismatch repair-deficient tumors benefit from anti-PD-1 therapy 31. Further, prospective studies in large populations are required to assess the clinical utility of the custom panel.

In conclusion, we have developed a highly sensitive and specific method for the early detection of NSCLC. Our study highlights the potential clinical utility of the combined assay results and post-operative new ctDNA mutation status as prognostic indicators in NSCLC patients.

## Supplementary Material

Supplementary figures.

Supplementary tables.

## Figures and Tables

**Table 1:** Characteristics and demographics of 147 NSCLC patients

Characteristics	Patients
**Sex, n (%)**	
Male	99 (67.34%)
Female	48 (32.65%)
**Age, n (%)**	
Mean (SD)	60.03 (9.17)
Median (range)	62 (26-80)
**NSCLC subtype, n (%)**	
Adenocarcinoma (AC)	78 (53.06%)
Squamous cell carcinoma (SqCC)	56 (38.10%)
Other	13 (8.84%)
**AJCC Stage, n (%)**	
IA	5 (3.40%)
IB	62 (42.18%)
IIA	7 (4.76%)
IIB	19 (12.93%)
IIIA	31 (21.09%)
IIIB	8 (5.44%)
IV	9 (6.12%)
Unknown	6 (4.08%)

NSCLC: non-small cell lung cancer; AJCC: American Joint Committee on Cancer.

**Table 2:** Proportion of patients detected with each individual biomarker assay and combined assay

Assay type	Patients detected (n)							
	Type of NSCLC				AJCC Stage			
	AC (46)	SqCC (35)	Other (6)	NSCLC (87)	I (43)	II (14)	IIIA (20)	IIIB/IV (8)
EGFR (%)	24 (52.2)	11 (31.4)	3 (50.0)	38 (43.7)	20 (46.5)	4 (28.6)	9 (45.0)	3 (37.5)
KRAS (%)	6 (13.0)	1 (2.9)	1 (16.7)	8 (9.2)	4 (9.3)	2 (14.3)	1 (5.0)	1 (12.5)
ctDNA (%)	30 (65.2)	12 (34.3)	4 (66.7)	46 (52.9)	24 (55.8)	6 (42.9)	10 (50.0)	4 (50.0)
CEA (%)	7 (15.2)	2 (5.7)	0 (0)	9 (10.3)	5 (11.6)	1 (7.1)	3 (15.0)	0 (0)
CYFRA21-1 (%)	5 (10.9)	18 (51.4)	2 (33.3)	25 (28.7)	9 (20.9)	7 (50.0)	7 (35.0)	2 (25.0)
SCC (%)	4 (8.7)	7 (20.0)	0 (0)	11 (12.6)	2 (4.7)	4 (28.6)	3 (15.0)	1 (12.5)
Protein biomarkers (%)	12 (26.1)	21 (60.0)	2 (33.3)	35 (40.2)	24 (55.8)	9 (64.3)	9 (45.0)	2 (25.0)
Combined assay (%)	34 (73.9)	26 (74.3)	5 (83.3)	65 (74.7)	32 (74.4)	11 (78.6)	15 (75.0)	5 (62.5)

Two of the 87 NSCLC patients were missing AJCC Stage information. NSCLC: non-small cell lung cancer; AC: adenocarcinoma; SqCC: squamous cell carcinoma; CEA: carcinoembryonic antigen; CYFRA21-1: fragments of cytokeratin 19; SCC: squamous cell carcinoma antigen.

**Table 3:** Recurrence-free survival analysis by clinicopathologic variables and combined assay

Variable	Univariate analysis		Multivariate analysis	
	HR (95% CI)	*p*-value	HR (95% CI)	*p*-value
Age, ≥54 vs <54	0.49 (0.18-1.3)	0.16		
Sex, female vs male	1.1 (0.45-2.9)	0.78		
Type, Other vs SqCC vs AC	1.1 (0.54-2.2)	0.79		
AJCC Stage, III/IV vs I/II	5.8 (2.4-14)	< 0.001	7.6 (2.9-20)	< 0.001
Lymph node invasion, yes vs no	1.5 (0.64-3.6)	0.35		
Combined assay, positive vs negative	0.47 (0.2-1.1)	0.087	0.30 (0.12-0.77)	0.012

HR: hazard ratio; CI: confidence interval.

**Table 4:** Overall survival analysis by clinicopathologic variables and post-operative new ctDNA mutation status

Variable	Univariate analysis		Multivariate analysis	
	HR (95% CI)	*p-*value	HR (95% CI)	*p*-value
Age, ≥55 vs <55	0.59 (0.15-2.4)	0.45		
Sex, female vs male	0.33 (0.041-2.7)	0.30		
Type, Other vs SqCC vs AC	1.3 (0.45-3.7)	0.62		
AJCC Stage, III/IV vs I/II	5.3 (1.3-22)	0.023	16.8 (1.8-155)	0.013
Lymph node invasion, yes vs no	1 (0.21-5.1)	0.97		
Post-operative new ctDNA, positive vs negative	2.6 (0.62-11)	0.19	10.5 (1.1-101)	0.042

HR: hazard ratio; CI: confidence interval.

**Table 5:** Recurrence-free survival analysis by clinicopathologic variables and post-operative new ctDNA mutation status

Variable	Univariate analysis		Multivariate analysis	
	HR (95% CI)	*p-*value	HR (95% CI)	*p*-value
Age, ≥55 vs <55	0.59 (0.15-2.4)	0.46		
Sex, female vs male	0.36 (0.045-3.0)	0.34		
Type, Other vs SqCC vs AC	1.3 (0.44-3.6)	0.66		
AJCC Stage, III/IV vs I/II	4.3 (1.0-18)	0.045	13 (1.5-128)	0.020
Lymph node invasion, yes vs no	0.97 (0.20-4.8)	0.97		
Post-operative new ctDNA, positive vs negative	2.8 (0.66-12)	0.16	11 (1.1-108)	0.038

HR: hazard ratio; CI: confidence interval.

## Abbreviations

NSCLC: non-small cell lung cancer
ctDNA: circulating tumor DNA
cfDNA: cell-free DNA
tDNA: tumor DNA
*EGFR*: epidermal growth factor receptor
*KRAS*: Kirsten rat sarcoma viral oncogene homolog
NGS: next-generation sequencing
VAF: variant allele frequency
AC: adenocarcinoma
SqCC: squamous cell carcinoma
CEA: carcinoembryonic antigen
CYFRA21-1: fragments of cytokeratin 19
SCC: squamous cell carcinoma antigen
AJCC: American Joint Committee on Cancer
RFS: recurrence-free survival
OS: overall survival
ROC: receiver operator characteristic
HR: hazard ratio
CI: confidence interval
