# Supplementary material for: Combined assay of Circulating Tumor DNA and Protein Biomarkers for early noninvasive detection and prognosis of Non-Small Cell Lung Cancer
Source: J Cancer. 2021 Jan 1;12(4):1258–69. doi: 10.7150/jca.49647 (PMC7797658; doi:10.7150/jca.49647)
Supplement: Supplementary file 1 — Supplementary figures. [file jcav12p1258s1.pdf]

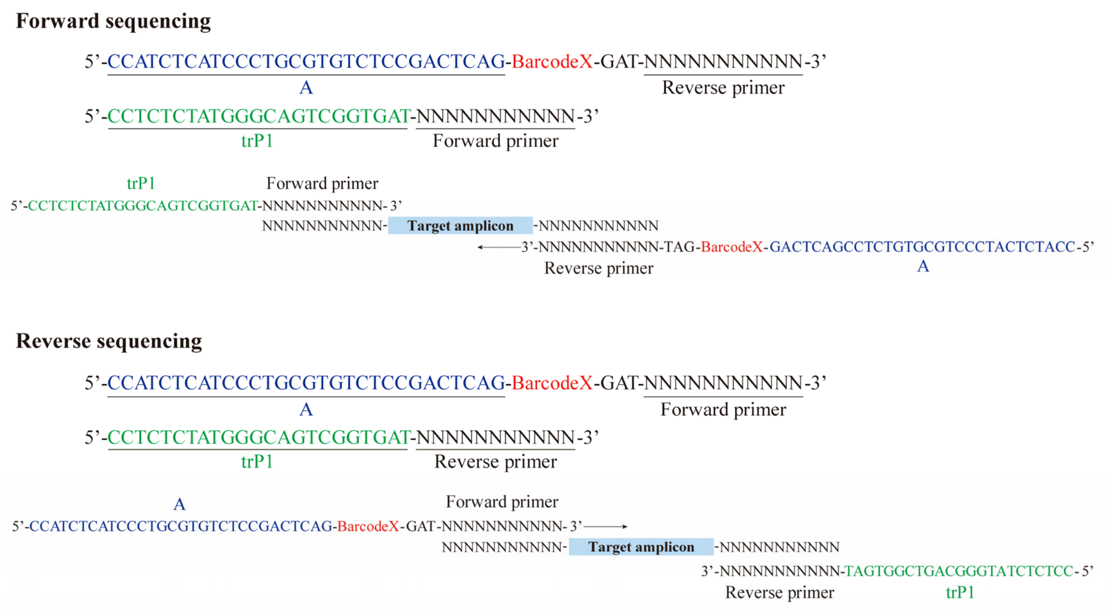

S1 Fig. Example fusion primers design. Each amplicon requires two pairs of fusion primers for bidirectional sequencing. The barcode sequence in the primer is used to identify different samples. Both A and trP1 are adaptor sequences.
